# The Influence of Place of Residence, Gender and Age Influence on Food Group Choices in the Spanish Population: Findings from the ANIBES Study

**DOI:** 10.3390/nu10040392

**Published:** 2018-03-22

**Authors:** María de Lourdes Samaniego-Vaesken, Teresa Partearroyo, Emma Ruiz, Javier Aranceta-Bartrina, Ángel Gil, Marcela González-Gross, Rosa M. Ortega, Lluis Serra-Majem, Gregorio Varela-Moreiras

**Affiliations:** 1 Department of Pharmaceutical and Health Sciences, Faculty of Pharmacy, CEU San Pablo University, 28668 Madrid, Spain; l.samaniego@ceu.es (M.d.L.S.-V.); t.partearroyo@ceu.es (T.P.); 2 Spanish Nutrition Foundation (FEN), 28010 Madrid, Spain; eruiz@fen.org.es; 3 Department of Preventive Medicine and Public Health, University of Navarra, 31008 Pamplona, Spain; jaranceta@unav.es; 4 CIBEROBN, Biomedical Research Networking Center for Physiopathology of Obesity and Nutrition, Carlos III Health Institute, 28029 Madrid, Spain; agil@ugr.es (Á.G.); marcela.gonzalez.gross@upm.es (M.G.-G.); lluis.serra@ulpgc.es (L.S.-M.); 5 Department of Biochemistry and Molecular Biology II, Institute of Nutrition and Food Sciences, University of Granada, 18010 Granada, Spain; 6 ImFINE Research Group, Department of Health and Human Performance, Universidad Politécnica de Madrid, 28040 Madrid, Spain; 7 Department of Nutrition, Faculty of Pharmacy, Madrid Complutense University, 28040 Madrid, Spain; rortega@ucm.es; 8 Research Institute of Biomedical and Health Sciences, University of Las Palmas de Gran Canaria, 35016 Las Palmas de Gran Canaria, Spain

**Keywords:** socioeconomic factors, place of residence, habitat size, food group consumption, intake, ANIBES study

## Abstract

Socioeconomic factors (SEF) can exert a great impact on food choices. However, limited data are available from the Spanish population. Our aim was to describe the influence of place of residence and habitat size on food group intakes. Data were obtained from the ANIBES study. A 3-day dietary record provided information on food and beverage consumption. Data analysis compared gender, age, Nielsen geographic areas, and habitat population size (urban, semi-urban, and rural). Place of residence did not appear to be a determinant for specific food group consumption during childhood and adolescence, as only higher intakes of non-alcoholic beverages were observed among children aged 9 to 12 years living in the East, when compared to those from the Northwest of Spain (*p* < 0.05). Food choices within adults (18 to 64 years) and seniors (65 to 75 years) were conditioned: sugar and sweets intake was significantly higher (*p* < 0.05) for adult men living in the Northwest than those from the South, and senior males from North Central areas had significantly higher consumption of eggs (*p* < 0.05) compared to the Northeast. Basic food group consumption was only affected during childhood and aging. Adults who inhabited rural areas consumed greater quantities of fats and oils than those from higher population densities (*p* < 0.01). Our results indicate that place of residence and habitat size have a limited influence on food choices, regardless of age and gender in the ANIBES study population. It is fundamental to acknowledge that other SEF variables are important and further studies are needed to monitor and assess these influences are warranted.

## 1. Introduction

It is widely recognized that food choices, which affect diet composition, can be a determinant in the health and disease status of a population. Throughout the last five decades, researchers, organizations, and public health authorities have emphasized the importance of a varied, balanced, and moderated diet to reduce the risk and burden of diet-related chronic diseases, such as cardiovascular disease, type 2 diabetes, some cancers, and obesity [1,2,3]. Recently published data on overweight and obesity prevalence in Spain [4,5], indicated an alarming rate increase, with the prevalence of overweight being 35.8% and obesity 19.9%. Although several programmes have been implemented by government and health authorities in our country [6], the current scenario does not seem to be improving in children, adolescents [7], or adults [4]. A similar situation has been described in other European countries (i.e., Belgium and France) [8]. Reducing health-related costs, as well as improving the present and future quality of life of individuals, is at stake. In 2014, the World Health Organization Global status report on non-communicable diseases underlined the urgent need to decrease the overweight and obesity burden and the path forward to improve dietary habits by developing specific targets, such as policies for the sugar, salt, and fat reduction in food products [9].

Inequalities or disparities derived from socioeconomic factors (SEF) were proposed to have an influence on the dietary and health habits of individuals and, at present, are considered a matter of great concern [2,3,10]. Researchers recommended the development of integral studies when looking at the energy balance equation, as the interrelationship of all influential variables should be accounted for globally [1]. Conversely, they acknowledged that technological advances and improvements in socioeconomic conditions (i.e., better acclimatization conditions in houses and workplaces, mechanization of labor tasks, improvement in public transportation, and increase in the use of private transportation) are closely linked to profound transformations in dietary choices, together with increased sedentary time and a reduction in physical activity levels [1]. Looking at the *whole picture* can help authorities develop strategies for tackling this widespread problem. A recent systematic analysis by Forouzanfar et al. [3] concluded that behavioral, environmental, occupational, and metabolic risks (i.e., high body mass index) can explain half of total global mortality and more than one-third of global disability-adjusted life years (DALYs), thus providing many opportunities for prevention.

Many factors have been shown to influence our food selection and dietary habits, including geographical situation or place of residence, cultural and intrapersonal variables, and other SEFs, such as education and income [3]. Nevertheless, only few studies describe the present situation in our country [11,12,13,14]. What we do know, is that the Spanish diet has slowly but continuously drifted away from the Mediterranean dietary pattern over the last two decades, and this pattern is recognized as the healthy model to pursue [15,16,17]. 

One of the specific goals of the ANIBES study (anthropometric data, macronutrients and micronutrients intake, practice of physical activity, socioeconomic data and lifestyles) was to accomplish an update of the main food and beverage group/subgroup intake and their contribution to the total energy intakes and dietary habits of the Spanish population (9–75 years) [18]. In this regard, previous work from the ANIBES research group has reported several important issues. Results showed that bread (11.9%), olive oil (9.6%), and meat (8.9%) were the top three energy providers within the ANIBES population (m/f, 9–75 years) from all the food subgroups studied [19]. Total energy intake among men and women presented a substantial decrease when compared to intakes from 1964 (3008 kcal/person/day) and 2010 (2609 kcal/person/day) [19]. There was also an unbalanced macronutrient distribution in the diet of the overall population, where there was a higher total fat intake of 37.9% (compared to European Food Safety Authority (EFSA) recommendations of 20–35%), lower carbohydrate intake of 41.4% (EFSA: 45–60%), and protein accounted for 17% of total energy (EFSA: 12–15%). However, one positive aspect was the high monounsaturated fatty acid intake, due to olive oil consumption [20]. A low adherence to the Dietary Guidelines in Spain, especially in the overweight/obese population was observed in the analysis by Rodríguez-Rodríguez et al. [21]. Finally, the clustered lifestyle patterns and diet quality of the ANIBES population sample were analyzed by Perez-Rodrigo et al. [22], which showed that a major proportion of people aged 18–30 years could be classified within the “*Poor diet-low physical activity-sedentary lifestyle pattern*”.

Our working hypothesis was that SEFs, such as area of residence (Nielsen areas) and habitat size (urban, semi-urban, and rural), may play a role in influencing food availability, choice, selection, purchasing, and therefore, intakes among the Spanish population. This, in turn, could affect global diet and nutrient intakes. In the present study, our objective was to analyze and describe the relationship between these two SEF variables and the ANIBES study population’s food group intake. In consequence, we aimed to assess food group consumption in relation to relevant SEFs, namely geographical area of residence (Nielsen areas) and habitat size, from different age groups of participants from the ANIBES study, as a representative sample of the Spanish population.

## 2. Materials and Methods

The complete design, protocol, and methodology of the ANIBES study have been previously published in detail elsewhere [18,19,23]. The following paragraphs give a summary for comprehension of the present analysis. 

### 2.1. Sample

ANIBES was a cross-sectional study, conducted using stratified multistage sampling. The fieldwork was performed at 128 sampling points across Spain from mid-September 2013 to November 2013 (three months). The study protocol was approved by the Ethical Committee for Clinical Research of the Madrid Region, Spain, coded as “FEN 2013”, and approved on 31 May 2013 [18]. The study design aimed to define a representative sample size of all individuals living in Spain (excluding Melilla and Ceuta autonomous cities in North Africa), aged 9 to 75 years, and living in municipalities of at least 2000 inhabitants. The final sample consisted of 2009 individuals (1013 men, 50.4%; 996 women, 49.6%) and a boost sample that was included for the youngest age groups (9–12, 13–17, and 18–24 years), to have at least *n* = 200 per age group (error +/− 6.9%). Therefore, the random sample plus the booster sample consisted of 2285 participants. Further investigation into physical activity patterns and behaviors from the ANIBES population can be reviewed in previous study publications [24,25,26], as described for children and adolescents [24,26], as well as adults [24,27] and in relation to other lifestyle variables, such as smoking and sleeping habits [27]. Exclusion criteria and eligible individuals were described in detail by Ruiz et al. [18]. Trained interviewers collected all data and participants provided written informed consent.

### 2.2. Socioeconomic Factors: Demographic Data and Area of Residence

Participants answered a face-to-face, validated questionnaire [18] that included the following questions: age in completed years (9 to 12, 13 to 17, 18 to 64, and 65 to 75 years), place of birth (no immigrant/immigrant), educational level (primary or less/secondary/university), occupational status (unemployed/employed), and monthly family income (0–1000€/1001–2000€/ > 2000€/no answer). Validation was performed in two pilots studies, as detailed in the study design and methodology publication [18].

**Figure 1:**
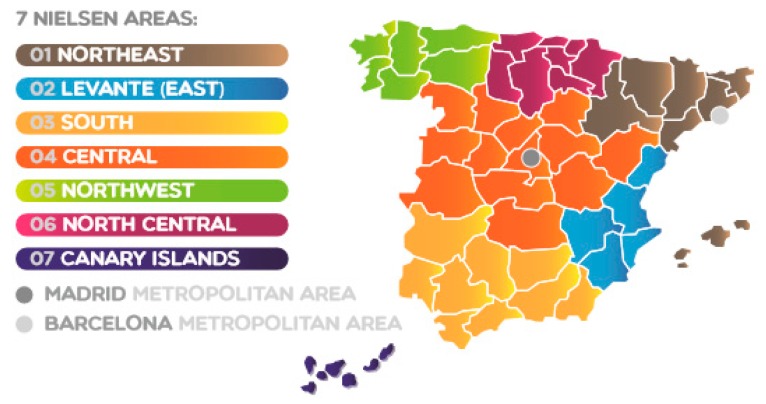
Nielsen area distribution in the Spanish territory.

Geographical distribution was structured according to the Nielsen area scheme, as previously described in Ruiz et al. [18], and included Barcelona (Metropolitan area), the Canary Islands, Central, Levante (East), Madrid (Metropolitan Area), Northeast, Northwest, North Central and South (Figure 1). Locality or habitat size classes were 2000 to 30,000 inhabitants (rural population), 30,000 to 200,000 inhabitants (semi-urban population), and over 200,000 inhabitants (urban population).

Relative distribution ranges of our sample across the Nielsen areas are presented in Table 1.

### 2.3. Dietary Assessment

Dietary intake was assessed by means of a face-to-face, 24-h diet recall interview assisted by a food picture atlas to estimate portion sizes. In addition, participants completed a three-day food record by using a tablet device (Samsung Galaxy Tab 27.0, Samsung Electronics, Suwon, South Korea). Two consecutive weekdays and one weekend day recording all foods and beverages consumed at home and away from home were included. Processing of food record inputs, coding, and data cleaning is reported elsewhere. Energy and nutrient intakes were calculated using software specifically developed for the ANIBES study (VD-FEN 2.1 software-Dietary Evaluation Programme, Spanish Nutrition Foundation, Madrid, Spain); this software was developed based on data from the Food Composition Tables by Moreiras et al. [28]. Food and beverages were arranged into 16 food groups (Supplementary Material Table S1), which comprised a total of 754 food items, grouped taking into account the similarities of their nutrient profiles [22], and consumption was expressed as grams per day.

### 2.4. Statistical Analysis 

Parametric data were statistically analyzed by a one-way Analysis of Variance (ANOVA). When the ANOVA resulted in differences, multiple comparisons between means were studied by Games–Howell or Student–Newman–Keuls tests. Values were expressed as the mean and standard error of the mean (SEM). Differences were considered significant at *p* < 0.05. Variables were tested for normality using a Kolmogorov-Smirnov test. Data analysis was performed with SPSS version 24.0 software package (IBM Corp., Armonk, NY, USA).

## 3. Results 

In the present study, our aim was to evaluate if there was significant variability in food group intake among geographical areas of Spain. Firstly, Table 2 and Table 3 show the global distribution of food group intake, expressed as grams per day, for the male and female population, respectively, segmented by Nielsen areas, and regardless of age. Non-alcoholic beverages (including water, coffee and teas, sugar-sweetened soft drinks, non-sweetened soft drinks, sports and energy drinks, and juices and nectars) were the main consumed group for both genders and all geographical areas. Next, milk and dairy products comprise the main food groups, as already described by Ruiz et al. [19,20]. The overall distribution of intake of food groups for the male and female populations, respectively, segmented by habitat size (urban, semi-urban, and rural of 2000–30,000, 30,000–200,000, and over 200,000 inhabitants, respectively), are shown in Table 4.

Results obtained from different age segments are presented as Supplementary Material tables (please refer to Tables S1–S8) and will be cited and analyzed throughout this section.

### 3.1. Children (9 to 12 Years)

Consumption of the sixteen major food groups among the children population (boys and girls) from the ANIBES study were similar when assessed by geographical area (Nielsen areas). It is noteworthy that our results showed boys living in the East of Spain drank a significant higher quantity of non-alcoholic beverages (*p* < 0.05) than those living in the Northwest regions. Moreover, although higher intake levels could be observed for children from the Canary Islands, these values were not significant (Table S1).

From Table S2, we can see that boys living in semi-urban and urban areas consume significantly higher quantities of meat and meat products (*p* < 0.05) than those from rural zones, with 171.1 and 177.1 g/day, respectively. In addition, they have higher intakes of milk and dairy products (*p* < 0.05) when compared to their rural counterparts. Remarkably, we found no differences in food group intakes among girls, regardless of habitat.

### 3.2. Adolescents (13 to 17 Years)

Within this group, we also failed to find significant differences in the consumption of any of the studied food groups and across the nine Nielsen areas (Table S3). However, when studying habitat size, we found that adolescent girls living in rural areas had a significant lower intake of ready-to-eat meals (*p* < 0.01) than those from semi-urban areas. In addition, data provided in Table S4 displays that rural boys consumed a significant higher amount of cereals and grains (203.1 g/day, *p* < 0.05) than their peers from urban sites (160.8 g/day).

### 3.3. Adults (18 to 64 Years)

Men living in the South of Spain had significantly higher intakes of appetizers (i.e., olives with/without bone, pretzels, popcorn with/without oil and salt, potato chips, and pickled cucumber) (*p* < 0.05) than those living in Madrid and the Northwest. Sugar and sweets intake was significantly higher (*p* < 0.05) for men living in the Northwest than those from the South. It is also remarkable that meat consumption among men living in the Northcentral area was significantly higher (*p* < 0.01) than intakes of those from the Northwest. Likewise, we observed a significantly higher intake of eggs (*p* < 0.05) among men from Central Spain when compared with those living in the Eastern areas. Nevertheless, men living in the former area had significantly lower milk and dairy product intake (*p* < 0.05) when compared to their Madrid (metropolitan area) peers (Table S5).

In Table S6, intake of food groups among adults is compared by habitat size. We found that men living in rural and semi-urban areas had higher intakes of oils and fats (*p* < 0.01) than those from urban areas. Furthermore, the former had higher appetizer (*p* < 0.05) and legume intakes (*p* < 0.05), as well as higher alcoholic beverage consumption (*p* < 0.05), than those living in semi-urban settings. On the other hand, milk and dairy product intake was lower (*p* < 0.01) among men living in semi-urban areas. Finally, supplements and ready-to-eat meals were lower in men from urban areas (*p* < 0.05) when compared to those from semi-urban zones.

In the female group, we observed that fat and oil intakes were significantly higher in women from the South when compared to intakes from those living in the Canary Islands (*p* < 0.01), Central (*p* < 0.01), East (*p* < 0.01), Madrid (metropolitan area) (*p* < 0.01), and Northwest (*p* < 0.01) areas. Although, appetizer consumption in women living in Madrid (metropolitan area) (*p* < 0.01) and the Canary Islands (*p* < 0.05) were lower when compared with intakes from women living in East area. On the other hand, it is noteworthy that intake of alcoholic beverages was significantly lower among women living in Barcelona (Metropolitan area) than in those living in the Central (*p* < 0.01), East (*p* < 0.01), or South regions of the Peninsula (*p* < 0.01). Non-alcoholic beverages were mainly consumed amongst women living at the Northwest (*p* < 0.001) and South (*p* < 0.001), compared to those living in East areas (Table S5). Results for adult women did not present any significant differences when evaluated according to their habitat of residence (Table S6). 

### 3.4. Seniors (65 to 75 Years)

When food group intakes from the senior male population were examined (Table S7), we found a significantly higher consumption of eggs among those living in North Central (*p* < 0.05) when compared to individuals living in Northeast areas. In addition, vegetable intake from the former group was higher (*p* < 0.05) than intake from Southern senior males.

Senior women from the south of Spain consumed higher quantities of oils and fats (*p* < 0.05) than those in the Canary Islands. Likewise, their egg consumption was higher (*p* < 0.05) than in the Eastern area. Finally, vegetable group intake was lower (*p* < 0.05) among women from the South and Madrid Metropolitan areas when compared to those living in the North Central locations of Spain.

Senior men had a lower intake of pulses when living in big cities (urban) than when they inhabited semi-urban (*p* < 0.01) or rural (*p* < 0.01) zones. Nonetheless, this was not observed in senior women (Table S8).

## 4. Discussion

### 4.1. Potential Factors Affecting Food Choice: Food Groups and Geographical Disparities in Spanish Geography

A few decades ago, it was generally acknowledged that dietary habits encompassing the food choices of the population were deeply influenced by the geographic areas and places of residence considered [29,30]. These factors were also linked to socioeconomic status and would affect food availability and accessibility for determined population groups, namely those with fewer economic resources. The classic study by Covián et al. [29], in 1964, was among the first to show the influence and relevance of geographic and economic disparities on malnutrition in a group of schoolchildren from a poor peripheral district of Madrid. However, it is a fact that studies conducted in the last decade seem to show that these differences have started to dilute [11]. Demographic variables and their influence on food product availability and accessibility have been analyzed in a number of studies internationally [31], in Europe [17], and in our country [22,26], with most researchers agreeing that, at present, these matters seem to have been overcome in most urban and semi-urban areas of Spain. Globalized markets that encompass increased, regular transportation, distribution, and homogeneous commercialization of a high variety of food staples, regardless of their origin, are factors enabling diet variety and choice. Our results are in line with these premises and indicate that urban and rural differences in food purchasing and consumption, at present, could be considered as moderate. 

Food Consumption Panel series data from the Spanish Ministry of Agriculture and Fisheries, Food and Environment (MAPAMA), has reflected a marked and continuous increase in meat product consumption and a decrease in the consumption of pulses and legumes. Food prices are potentially a major determinant for consumer purchase decisions, but according to data from the MAPAMA, in the last decade, although a decrease in prices was observed during the recent economic crisis (2008–2014), other, somewhat expensive food groups have been stable in their consumption. Nevertheless, this crisis deeply affected the dietary habits of important sectors of the population, for example, among the infant population, as revealed by Antentas et al. [32]. In 1998, García et al. [33] analyzed differences in the behavior of rural and urban consumers from 2500 nationwide households when purchasing food products. Their main findings highlighted how different price levels existing in each locality type accounted for the main significant differences in food acquisition patterns. They also underlined that income level was a determinant for food choices only when comparing between rural and urban areas and not when comparing urban locations.

Although several studies show that the Spanish population has a marked tendency to abandon Mediterranean diet patterns (MDP), some have divergent results. For instance, in the Balearic adult population, a study by Bibiloni et al. found a stable situation and even a moderate recovery between 1999 and 2010 [34]. The ANIBES study also found some significant results regarding the maintenance of healthy habits, like the consumption of olive oil as the main dietary fat, which improves lipid profile quality [20]. In the next paragraphs, we assess the intakes of some food groups included in the MDP according to our results.

Fish and shellfish consumption was traditionally described as higher through the coastal areas of our country in the 90s; however, it has been markedly increasing in the Spanish food basket over the past few decades, according to data from the Food Consumption Panel series [35]. Consumption has been shown to be higher among elderly individuals of higher socioeconomic status and in urban areas [32,35,36]. At present, however, consumption seems to be equally distributed across Central and Northwest when compared to Northern, Southern, or even the Canary Islands, where fish and shellfish intakes are not significantly different in the present study. Higher intakes were only found in male seniors from Central, Madrid, and Northeast areas (Table S7), although these differences were not significant. Likewise, we found no significant differences in Metropolitan areas of Barcelona and Madrid, where food distribution is allegedly improved. However, for this food group, female adolescents from the two capital cities presented increased (but not significant) intakes of 98.7 (139.5) and 95.5 (97.4) g/day, respectively. Concerning habitat size, we cannot report any significant differences throughout the study sample.

The dietary habits and food choices of younger groups in our country is a controversial issue, namely for fruits and vegetables, as intakes of these food groups have been reported as far from sufficient to meet dietary guidelines [37]. Results from enKid [38], carried out between 1998 and 2000, in a nationwide sample of 1629 boys and 1905 girls (*n* = 3534, 2–24 years), indicated that 47% (95% confidence interval 46–48%) of the sample disliked vegetables and an additional 5.7% (95% confidence interval 4.9–6.5%) disliked fruits. The authors concluded that there was a significant relationship between the like or dislike for fruits and vegetables and the consumption of these food groups. We did not observe any significant differences between adults, children or adolescents fruit and vegetable consumption, considering their geographical location or place of residence. In contrast, we found that fruit and vegetable consumption was higher for the senior group. Other previous studies corroborate these findings and how the elderly in our country have better dietary habits, in line with the MDP [13].

The pulses or legumes group showed no significant differences across geographical areas or place of residence. Many recent publications [39,40] discuss that current intakes from the Spanish population are considerably lower than those of our ancestors. We did not find any significant differences in consumption in the studied geographic areas. In the past, their consumption has been associated with lower socio-economic classes, and in fact, pulses were known as “*the protein from poor*” [41]. Today, many beneficial aspects of their consumption are acknowledged, not only as a rich vegetable protein source, but also in terms of cardiovascular [42], weight management, and digestive health [43,44]. 

### 4.2. Consumption of Specific Food Groups Which Should Be Moderate According to Spanish Dietary Guidelines

The offer and intake of ready-to-eat meals has been continuously growing in recent years. Convenience, price, ease of use, and lack of time for cooking are related to this phenomena in Western societies. We would have expected to find higher intakes amid Metropolitan and urban areas, but this was not the case. In fact, when assessing intakes according to habitat size, we found that adolescent girls from rural environments (*p* < 0.01) and adult men from urban areas (*p* < 0.05) had lower intakes than their semi-urban counterparts. Spanish society seems reluctant to abandon “*homemade*” dishes and typical foods from our rich gastronomy. Even today, restaurant and fast food companies acknowledge the need to commercialize food products that are typical of the regional gastronomy. This does not mean that other types of ready-to-eat meals are not gaining a market share; data from on-line food delivery companies in Spain show a marked increase in sales, especially among younger population groups, as leisure time habits are quickly changing and home purchases are increasing [45].

In the case of sugar and sweets, although there is a specific group comprising products that are high in free sugars, we have to bear in mind that this ingredient has widespread inclusion in processed food products, such as ready-to-eat meals, bakery, confectionery, and breakfast cereals, to name a few. The World Health Organization’s guidelines on sugar intake for adults and children, published in 2015, strongly recommend a reduction in free sugar intake for both adults and children, to less than 10% of total energy intake (EI), remarking that an additional reduction below 5% of total EI would provide further health benefits [46]. Results from our study fail to show significant differences between Nielsen areas and place of residence in most age and gender groups. The exception was adult males from the Northwest when compared to their Southern counterparts. However, other studies have found higher added sugar consumption among adults from rural areas and that subjects who consumed ≥10% energy from added sugars had a higher waist circumference and body mass index [47]. Ruiz et al. [48] evaluated intake and key food and beverage sources of total and free sugars in the ANIBES sample. They found that median total sugar intake was 71.5 g/day (17% Total Energy, TE), intrinsic sugar intake was 38.3 g/day (9.6% TE), and free sugar intake was 28.8 g/day (7.3% TE). The three main sources of intrinsic sugars were fruits (31.8%), milks (19.6%), juices and nectars (11.1%), and in the case of free sugars, sweetened soft drinks (25.5%), added sugar (17.8%), bakery and pastry items (15.2%), and chocolates (11.4%). The authors highlighted that only a moderate percentage of the Spanish population adhered to the present recommendations for total sugar intake, which is a matter of concern, especially among children and adolescents.

It is a fact that food group selection can greatly influence macronutrient intakes such as protein, fats and carbohydrates. Lipid profile, which is a major determinant in many chronic-degenerative diseases, is deeply modified depending on dietary habits that encompass food group selection [9,49]. The recently published DRECE study (*Diet and Risk of Cardiovascular Diseases in Spain*) prospectively evaluated the nutrient consumption and lipid profile of the diets of 4783 individuals by geographical area, between 1991 and 2010. Researchers found that the East and Southern areas of Spain had the highest fat intake, coupled with a high unhealthy lipid profile rate and cardiovascular mortality [12]. In addition, micronutrient intakes can be seriously compromised by poor or unbalanced dietary patterns, especially for those target population groups in which requirements are increased (i.e., women of childbearing age, children, and seniors). So far, no representative studies in Spain have analyzed the influence of place of residence and, globally, other SEFs on micronutrient intake and status. Results from the ANIBES study indicated that, globally, an elevated percentage of the population were not meeting the EFSA recommended intakes (RDIs ≤ 80%) for vitamin A (60%), vitamin E (80%), vitamin C (36%), selenium (25%), zinc (83%) [50], vitamin D (93%), calcium (66%), and magnesium (72%) [51]. It is noteworthy that in the case of iron intake, only 27.3% of women had RDIs above 80%, while more than 77% of men did [52]. In consequence, SEF variables must be taken into consideration in the future for a better understanding of these high and potentially insufficient intakes, targeting recommendations that are more precise and interventions at a population level.

### 4.3. Strengths and Limitations 

Ruiz et al. [18] discussed the problem of underreporting and its consistency across different surveys, and how the use of new methodologies to avoid this usual bias is challenging. In fact, when assessing food consumption, the potential recall bias introduced by respondents in reporting their diets is strongly associated with a tendency to overestimate food consumption accepted as healthy and underestimate the least healthy food. This must be taken into account [12]. It is crucial to improve the tools for studying and assessing the energy intakes and losses of “free living” independent subjects. In this regard, tools such as databases of the composition of quality foods, especially regarding energy and serving sizes should be improved, as stated at the recent Consensus Document and Conclusions on “*Obesity and Sedentarism in the 21st Century: What can be done and what must be done?*” [1]. The use of “new technologies” (i.e., tablet devices) was assessed and proved of great value in the present study. As expected, the senior population was the most reluctant to use and understand these tools, but data from Ruiz et al. [18] showed that the influence of this in final results was not significant. There is consensus that diet and food composition and consumption is still largely unknown in many respects, becoming even more complex with the inclusion of processed foods, and the wide variety of local foods and recipes [53]. Food composition data should be of a high quality, in order to assess nutrient intakes as accurately as possible. Many research projects are handling this work in our country and in Europe (i.e., EuroFIR and EFSA Foodex databases [54]). The ANIBES study methodology included a custom built food composition database to overcome the possible bias of using other food composition sources [18]. 

Some researchers argue that specific food groups include very heterogeneous subgroups, as is the case of non-alcoholic beverages, where water, juices, and nectars are together for data analysis in the present study. Another example pertains to vegetables, such as potatoes and other tubers, that might not have been included in this group. Other publications from the ANIBES group overcome this issue, for example the publication by Nissensohn et al. [55], in which non-alcoholic beverage consumption was assessed in detail to account for energy intakes from the many sources that are part of this food group. In the vegetable group, starchy tubers, such as potatoes, were included and this has been questioned in the literature previously, as their nutrient profiles are quite different. This can lead to over or underestimations related to the misclassification of food groups. 

The ANIBES study had several strengths, which included the careful design, protocol and methodology used, and that it was conducted among a random representative sample of the Spanish population aged 9 to 75 years. It was the first Spanish study at a national level that analyzed data for the whole population, as well as plausible reporters, as recommended by EFSA. One limitation of the study was its cross-sectional design, which provides evidence for associations but not causal relationships.

## 5. Conclusions

Our results indicate that in the ANIBES study population, socioeconomic factors comprising place of residence and habitat size have a limited influence on food group choices, although specific exceptions can be described for some gender and age groups. Nonetheless, it is fundamental to acknowledge that other socioeconomic variables are important, and further studies to continuously monitor, survey, and weigh these influences are required to develop effective and efficient dietary public health interventions and policies.

## Figures and Tables

**Table 1:** Relative distribution of the population sample from Nielsen areas.

	Total		Male		Female	
	*n*	%	*n*	%	*n*	%
**Northeast**	240	11.9	121	11.9	119	11.9
**Levante (East)**	335	16.7	176	17.4	159	16.0
**South**	443	22.1	218	21.5	225	22.6
**Central**	191	9.5	107	10.6	84	8.4
**Northwest**	152	7.6	77	7.6	75	7.5
**North Central**	162	8.1	80	7.9	82	8.2
**Canary Islands**	93	4.6	44	4.3	49	4.9
**Madrid Metropolitan Area**	264	13.1	133	13.1	131	13.2
**Barcelona Metropolitan Area**	129	6.4	57	5.6	72	7.2

**Table 2:** Intake of major food groups (g/day) across the male population in the ANIBES study, segmented by geographical area (Nielsen regions).

	Barcelona (Metropolitan Area)	Canary Islands	Central	Levante (East)	Madrid (Metropolitan Area)	Northeast	Northwest	North Central	South
***n***	57	44	107	176	133	121	77	80	218
**Oils and fats (g/day)**	24.1 ^a,b^ (9.7)	24.9 ^a,b^ (14.3)	25.3 ^a,b^ (11.7)	24.1 ^a,b^ (10.1)	22.1 ^a^ (9.4)	25.8 ^a,b^ (11.7)	23.1 ^a,b^ (12.9)	27.5 ^b^ (11.2)	26.0 ^a,b^ (12.7)
**Appetizers (g/day)**	5.6 (9.5)	5.9(10.8)	5.6 (16.3)	8.3 (13.0)	2.9 (6.7)	6.3 (11.5)	3.8 (9.3)	6.1 (13.1)	6.6 (14.9)
**Sugars and sweets (g/day)**	12.9 (11.8)	17.0 (15.4)	17.2 (17.2)	14.9 (16.1)	16.4 (14.2)	17.0 (19.0)	16.5 (16.7)	18.5 (19.4)	12.3 (13.9)
**Alcoholic beverages (g/day)**	94. (163.2)	151.4 (294.9)	110.1 (184.0)	154.9 (224.2)	114.0 (201.2)	88.2 (225.3)	130.0 (274.5)	121.2 (195.5)	186.0 (290.9)
**Non-alcoholic beverages (g/day)**	805.96 ^c,d^ (454.4)	788.3 ^c,d^ (409.7)	776.9 ^c,d^ (472.4)	977.3 ^d^ (630.7)	883.6 ^c,d^ (511.2)	851.8 ^c,d^ (509.6)	723.0 ^c^ (596.8)	950.1 ^c,d^ (527.4)	757.2 ^c,d^ (531.2)
**Meat and meat products (g/day)**	170.0 ^e,f^ (84.9)	146.1 ^e,g^ (80.2)	157.3 ^e,f,g^ (86.7)	172.3 ^e,f^ (88.1)	180.1 ^e,f^ (92.5)	179.7 ^e,f^ (90.1)	134.2 ^g^ (73.9)	187.2 ^g^ (87.8)	152.4 ^e,f,g^ (85.9)
**Cereals/grains (g/day)**	172.0 (73.6)	166.4 (55.6)	164.7 (65.4)	173.3 (71.6)	163.6 (71.8)	175.9 (73.8)	149.2 (66.8)	155.4 (64.9)	154.8 (60.8)
**Fruits (g/day)**	153.9 ^h,i^ (151.7)	216.7 ^i^ (236.2)	151.9 ^h,i^ (170.0)	161.8 ^h,i^ (170.8)	153.7 ^h,i^ (193.4)	145.9 ^h,i^ (178.7)	122.9 ^h^ (159.0)	193.1 ^h,i^ (200.3)	117.0 ^h^ (139.4)
**Eggs (g/day)**	27.0 ^j^ (27.4)	31.8 ^j,k^ (26.5)	38.4 ^j,k^ (31.6)	29.1 ^j,k^ (31.0)	31.4 ^j,k^ (30.1)	33.4 ^j,k^ (45.5)	32.1 ^j,k^ (32.4)	43.5 ^k^ (34.2)	31.7 ^j,k^ (29.4)
**Milk and dairy products (g/day)**	233.1 (165.6)	310.6 (219.8)	267.2 (141.6)	245.3 (193.6)	288.6 (164.2)	256.8 (191.5)	248.0 (161.0)	283.4 (152.8)	237.5 (152.9)
**Pulses (g/day)**	10.7 (14.6)	15.7 (19.2)	15.8 (20.9)	10.2 (14.5)	14.9 (21.9)	15.1 (21.89	13.3 (16.5)	15.1 (18.7)	16.0 (18.2)
**Fish and shellfish (g/day)**	72.7 (84.7)	45.4 (63.9)	62.4 (61.9)	64.3 (67.7)	56.0 (60.2)	49.8 (67.1)	72.9 (79.6)	70.5 (80.7)	58.4 (62.6)
**Ready-to-eat meals (g/day)**	89.6 (90.8)	76.2 (102.5)	77.9 (94.5)	75.9 (86.0)	88.2 (86.6)	91.6 (99.8)	65.9 (81.7)	60.5 (69.3)	80.6 (74.7)
**Sauces and condiments (g/day)**	15.6 (17.9)	11.4 (11.4)	12.9 (12.7)	17.5 (16.9)	14.0 (13.3)	15.2 (18.5)	11.3 (11.5)	13.8 (14.7)	14.1 (15.3)
**Supplements and meal replacements (g/day)**	0.1 (0.9)	1.7 (6.1)	0.5 (3.3)	0.0 (0.5)	0.0 (0.0)	1.5 (13.1)	0.2 (1.2)	0.3 (1.8)	0.5 (3.6)
**Vegetables (g/day)**	165.7 (94.2)	194.9 (112.2)	178.4 (114.9)	176.3 (120.8)	161.9 (101.4)	195.0 (134.8)	172.4 (107.8)	208.4 (142.8)	157.7 (104.0)

Data reported as means (standard error of the mean, SEM) per group. All differences are *p* < 0.05 (Student–Newman–Keuls test). Different superscript lowercase letters indicate statistical significance in each row.

**Table 3:** Intake of major food groups (g/day) across the female population in the ANIBES study, segmented by geographical area (Nielsen regions).

	Barcelona (Metropolitan Area)	Canary Islands	Central	Levante (East)	Madrid (Metropolitan Area)	Northeast	Northwest	North Central	South
***n***	72	49	84	159	131	119	75	82	225
**Oils and fats (g/day)**	25.7 ^a,b,c^ (11.5)	19.2 ^d^ (8.5)	21.9 ^a,d^ (10.5)	23.0 ^a,b,d^ (9.3)	22.8 ^a,b,d^ (11.1)	24.1 ^a,b,c^ (11.2)	21.8 ^a,d^ (7.9)	26.7 ^b,c^ (9.3)	27.6 ^c^ (12.1)
**Appetizers (g/day)**	5.8 (14.6)	4.1 (8.8)	4.3 (12.5)	7.1 (14.3)	2.0 (6.1)	6.0 (14.1)	3.8 (8.1)	6.4 (12.4)	5.2 (11.5)
**Sugars and sweets (g/day)**	13.4 ^e^ (12.8)	16.3 ^e,f^ (15.6)	20.1 ^e,f^ (19.1)	17.0 ^e,f^ (17.2)	14.0 ^e^ (13.7)	17.1 ^e,f^ (16.2)	18.1 ^e,f^ (15.5)	21.2 ^f^ (18.2)	14.1 ^e^ (14.6)
**Alcoholic beverages (g/day)**	28.1^g^ (53.3)	60.4 ^g,h^ (116.8)	82.9 ^g,h^ (135.4)	90.5 ^h^ (158.3)	46.9 ^g,h^ (144.6)	69.0 ^g,h^ (147.4)	40.8 ^g,h^ (80.2)	40.6 ^g,h^ (94.1)	66.8 ^g,h^ (131.0)
**Non-alcoholic beverages (g/day)**	838.4 ^i,j,k^ (496.4)	900.0 ^j,k^ (464.8)	843.7 ^i,j,k^ (444.7)	1042.9 ^k^ (523.8)	851.9 ^i,j,k^ (463.8)	934.3 ^j,k^ (591.2)	684.5 ^i^ (416.4)	880.0 ^i,j,k^ (592.0)	750.4 ^i,j^ (425.7)
**Meat and meat products (g/day)**	131.6 ^l^ (84.7)	94.2 ^m^ (59.0)	113.3 ^l,m^ (68.6)	123.7 ^l^ (68.4)	135.0 ^l^ (71.3)	125.1 ^l^ (76.0)	136.3 ^l^ (83.4)	137.2 ^l^ (71.6)	126.5 ^l^ (67.2)
**Cereals/grains (g/day)**	131.7 (54.0)	134.8 (51.2)	139.2 (58.4)	142.9 (57.4)	121.3 (51.5)	133.4 (57.3)	128.6 (62.1)	133.1 (56.4)	135.3 (51.4)
**Fruits (g/day)**	196.5 (159.2)	167.3 (182.0)	143.1 (144.1)	188.5 (178.0)	182.5 (176.8)	131.1 (156.1)	187.0 (196.1)	206.2 (264.4)	137.9 (145.8)
**Eggs (g/day)**	25.5 (28.3)	20.9 (21.8)	29.5 (25.3)	25.4(25.2)	25.1 (20.9)	23.0 (21.3)	30.6 (25.7)	30.4 (29.8)	23.9 (25.7)
**Milk and dairy products (g/day)**	230.2 ^n^ (123.1)	265.2 ^n,o^ (180.2)	267.0 ^n,o^ (144.6)	248.3 ^n^ (139.4)	272.2 ^n,o^ (147.7)	230.3 ^n,o^ (142.6)	268.1 ^n^ (160.6)	310.6 ^o^ (165.4)	244.3 ^n^ (137.0)
**Pulses (g/day)**	16.1 (20.1)	13.2 (18.4)	12.1 (14.4	12.4 (16.3)	11.9 (15.8)	11.0 (16.0)	15.0 (22.1)	11.6 (16.1)	13.3 (15.6)
**Fish and shellfish (g/day)**	88.2 ^p^ (88.0)	43.4 ^q^ (51.7)	60.2 ^p,q^ (95.6)	58.4 ^p,q^ (64.2)	69.8 ^p,q^ (71.0)	53.6 ^q^ (63.4)	66.6 ^p,q^ (66.3)	72.8 ^p,q^ (72.8)	63.7 ^p,q^ (68.0)
**Ready-to-eat meals (g/day)**	47.7 (67.7)	50.3 (53.0)	67.4 (70.4)	58.3 (63.8)	54.6 (59.3)	76.2 (75.0)	55.7 (57.1)	55.0 (61.6)	66.3 (71.5)
**Sauces and condiments (g/day)**	9.5 (10.0)	13.2 (11.0)	12.5 (15.4)	12.8 (15.1)	9.0 (11.7)	10.9 (12.5)	10.5 (14.9)	12.0 (13.6)	12.1 (13.9)
**Supplements and meal replacements (g/day)**	0.5 (2.0)	0.2 (1.4)	0.1 (1.1)	0.2 (1.6)	0.0 (0.0)	1.2 (10.8)	0.1 (0.8)	0.5 (3.4)	0.0 (0.2)
**Vegetables (g/day)**	212.6 ^r^ (125.3	181.7 (115.0)	156.4 ^r,s^ (106.9)	181.3 ^r,s^ (112.3)	175.7 ^s^ (102.6)	201.4 ^r,s^ (122.8)	176.0 ^r,s^ (98.3)	202.5 ^r,s^ (123.6)	162.8 ^s^ (91.3)

Data reported as means (standard error of the mean, SEM) per group. All differences are *p* < 0.05 (Student–Newman–Keuls test). Different superscript lowercase letters indicate statistical significance in each row.

**Table 4:** Food group intake (g/day) across the male and female population in the ANIBES study, segmented by habitat size (urban, semi-urban, and rural).

	Rural		Semi-Urban		Urban	
	Male	Female	Male	Female	Male	Female
***n***	345.0	337.0	358.0	337.0	325.0	334.0
**Oils and fats**	25.6 * (11.1)	24.7 (11.7)	25.7 * (12.0)	24.7 (9.6)	23.9 (11.2)	24.2 (11.1)
**Appetizers**	7.6 ^ ^ (13.9)	5.4 (11.8)	5.5 (12.2)	5.4 (12.1)	5.2 (11.3)	4.6 (11.9)
**Sugars and sweets**	15.4 (17.2)	18.1 ^+^ (16.8)	16.0 (16.1)	18.1 (16.9)	15.9 (14.9)	15.2 (14.0)
**Alcoholic beverages**	153.2 (269.4)	71.3 (146.2)	119.6 (204.6)	71.3 (97.5)	50.7 (235.9)	64.7 (141.7)
**Non-alcoholic beverages**	785.4 ^▪^ (522.0)	862.5 (555.4)	886.4 (538.0)	862.5 (477.3)	844.9 (559.7)	871.3 (465.7)
**Meat and meat products**	164.0 (89.9)	121.3 (69.3)	164.6 (83.0)	121.3 (72.8)	126.7 (91.0)	131.0 (74.4)
**Cereals/grains**	165.7 (64.4)	136.5 (53.8)	167.7 (70.5)	136.5 (56.1)	134.2 (68.4)	130.7 (56.0)
**Fruits**	154.1 (176.3)	162.8 (167.4)	151.5 (168.9)	162.8 (180.8)	162.7 (177.3)	173.9 (181.8)
**Eggs**	33.4 (36.9)	24.8 (24.7)	33.3 (29.5)	24.8 (24.3)	26.2 (31.7)	26.1 (25.9)
**Milk and dairy products**	242.3 (176.5)	255.5 (150.7)	271.0 (159.4)	255.5 (144.8)	269.0 (177.0)	244.2 (144.5)
**Pulses**	15.0 ^#^ (18.9)	13.0 (16.9)	15.3 ^#^ (20.4)	13.0 (15.8)	12.8 (16.3)	12.5 (17.6)
**Fish and shellfish**	59.1 (66.4)	61.7 (65.7)	65.0 (72.6)	61.7 (62.6)	60.2 (64.9)	69.7 (84.3)
**Ready to eat meal**	72.6 (79.7)	62.6 (65.0)	81.6 (90.1)	62.6 (67.0)	61.3 (88.2)	58.9 (67.6)
**Sauces and condiments**	13.6 (15.8)	11.1 (13.6)	14.0 (13.7)	11.1 (12.9)	12.1 (16.3)	11.1 (13.9)
**Supplements and meal replacements**	0.7 (8.1)	0.1 (0.7)	0.6 (3.4)	0.1 (2.2)	0.3 (0.8)	0.5 (6.5)
**Vegetables**	179.3 (121.0)	178.2 (107.9)	173.1 (119.6)	178.2 (106.5)	176.3 (106.0)	186.4 (114.2)

Data reported as means (standard error of the mean, SEM) per group. * *p* < 0.05 difference Urban (male) (Games–Howell test); ^ ^ *p* < 0.05 difference Urban (male) (Games–Howell test); **^▪^** *p* < 0.05 difference Semi-Urban (male) (Games–Howell test); ^#^ *p* < 0.05 difference Urban (male) (Games–Howell test); ^+^ *p* < 0.05 difference Urban (Games–Howell test).

## Supplementary Materials

The following are available online at http://www.mdpi.com/2072-6643/10/4/392/s1, Table S1: Food group classification and included subgroups assessed in the ANIBES Study, Table S2: Intake of major food groups (g/day) from the ANIBES Study amongst children (boys and girls, 9 to 12 years) segmented by geographical areas (Nielsen areas), Table S3: Intake of major food groups (g/day) from the ANIBES Study amongst children (boys and girls, 9 to 12 years) assessed by habitat size: rural, semi-urban and urban, Table S4: Intake of major food groups (g/day) from the ANIBES Study amongst adolescents (male/female, 13 to 17 years) segmented by geographical areas (Nielsen areas), Table S5: Intake of major food groups (g/day) from the ANIBES Study amongst adolescents (male/female, 13 to 17 years) assessed by habitat size: rural, semi-urban and urban, Table S6: Intake of major food groups (g/day) from the ANIBES Study amongst adult population (male/female, 18 to 64 years) segmented by geographical areas (Nielsen areas), Table S7: Intake of major food groups (g/day) from the ANIBES Study amongst adults (male/female, 18 to 64 years) assessed by habitat size: rural, semi-urban and urban, Table S8: Intake of major food groups (g/day) from the ANIBES Study amongst senior population (male/female, 65 to 75 years) segmented by geographical areas (Nielsen areas), Table S9: Intake of major food groups (g/day) from the ANIBES Study amongst seniors (male/female, 65 to 75 years) assessed by habitat size: rural, semi-urban and urban.
