# Supplementary material for: The Influence of Place of Residence, Gender and Age Influence on Food Group Choices in the Spanish Population: Findings from the ANIBES Study
Source: Nutrients. 2018 Mar 22;10(4):392. doi: 10.3390/nu10040392 (PMC5946177; doi:10.3390/nu10040392)
Supplement: Supplementary file 1 [file nutrients-10-00392-s001.pdf]

Supplementary Material

Table S1. Food group classification and included subgroups assessed in the ANIBES Study

(Adapted from Pérez-Rodrigo et al. (1))

Food group	Included subgroups
Cereals/Grains	Grains and flours<br>Bread<br>Breakfast cereals and cereal bars<br>Pasta<br>Bakery and pastry
Vegetables	Including starchy tubers (i.e. potatoes)
Fruits	
Oils and fats	Olive oil<br>Other oils<br>Butter, margarine and shortening
Milk and dairy products	Milk<br>Cheese<br>Yogurt and fermented milk<br>Other dairy products
Fish and Shellfish	
Meat and meat products	Meat
	Sausages and other meat products
	Viscera and spoils
Eggs	
Pulses	
Sugars and sweets	Sugar<br>Chocolates<br>Jams and other<br>Other sweets
Appetizers	
Ready-to-eat-meals	
Sauces and condiments	
Non-alcoholic beverages	Water<br>Coffee and infusions<br>Sugar soft drinks<br>Non-sweetened soft drinks<br>Sports Drinks<br>Energy drinks<br>Juices and nectars<br>Other drinks
Alcoholic beverages	Low alcohol content beverages<br>High alcohol content beverages
Supplements and meal replacements	

1. Pérez-Rodrigo C, Gianzo-Citores M, Gil Á, González-Gross M, Ortega R, Serra-Majem L, et al. Lifestyle Patterns and Weight Status in Spanish Adults: The ANIBES Study. Nutrients. 2017;9 (6):606.

Table S2. Intake of major food groups (g/day) from the ANIBES Study amongst children (boys and girls, 9 to 12 years) segmented by geographical areas (Nielsen areas)

	Barcelona (Metropolitan Area)		Canary Islands		Center		East		Madrid (Metropolitan Area)		Northeast		Northwest		North Central		South	
	Boys	*Girls*	*Boys*	*Girls*	*Boys*	*Girls*	*Boys*	*Girls*	*Boys*	*Girls*	*Boys*	*Girls*	*Boys*	*Girls*	*Boys*	*Girls*	*Boys*	*Girls*
**n**	6	2	7	5	10	9	21	14	12	9	15	10	9	6	10	14	36	18
**Oils and Fats (g/day)**	23.4 (6.8)	15.3 (3.3)	28.2 (13.0)	14.6 (4.8)	19.2 (9.6)	27.4 (11.4)	23.9 (6.3)	21.2 (9.8)	19.7 (10.2)	23.5 (5.6)	23.4 (10.0)	25.5 (9.4)	19.5 (11.0)	20.9 (6.5)	29.3 (9.6)	25.3 (7.0)	22.9 (10.9)	22.2 (9.6)
**Appetizers (g/day)**	4.2 (6.3)	17.3 (22.6)	15.8 (20.3)	14.5 (20.6)	0.2 (0.6)	1.1 (3.3)	5.1 (11.1)	2.8 (5.3)	3.6 (8.1)	6.1 (15.9)	2.7 (5.2)	0.3 (0.8)	3.0 (5.8)	6.5 (11.0)	3.6 (6.7)	4.7 (7.8)	6.7 (10.3)	7.2 (12.1)
**Sugars and sweets (g/day)**	16.6 (10.1)	30.3 (28.8)	32.7 (25.4)	6.9 (7.9)	19.8 (10.0)	21.8 (10.6)	27.1 (19.3)	20.1 (15.3)	24.5 (19.4)	29.9 (16.0)	35.4 (36.8)	28.7 (23.4)	16.9 (12.9)	24.3 (14.5)	21.0 (14.4)	33.1 (20.6)	21.9 (18.6)	23.5 (19.6)
**Alcoholic beverages (g/day)**	0.0 (0.0)	0.0 (0.0)	0.0 (0.0)	0.0 (0.0)	0.0 (0.0)	0.0 (0.0)	0.0 (0.0)	0.0 (0.0)	0.0 (0.0)	0.0 (0.0)	0.0 (0.0)	0.0 (0.0)	0.0 (0.0)	0.0 (0.0)	0.0 (0.0)	0.0 (0.0)	0.0 (0.0)	0.0 (0.0)
**Non-alcoholic beverages (g/day)**	786.1 (319.8)	525.0 (450.2)	1177.8 (594.7)	668.7 (201.4)	561.7 (310.2)	725.5 (347.5)	932.8* (477.3)	764.7 (524.9)	661.6 (462.2)	640.7 (305.9)	733.4 (332.7)	641.6 (360.5)	427.2 (303.9)	705.1 (186.6)	680.0 (354.4)	532.8 (352.9)	667.3 (421.9)	748.6 (376.5)
**Meat and meat products (g/day)**	224.4 (155.5)	107.5 (107.2)	132.4 (55.7)	92.0 (28.4)	153.4 (42.2)	137.7 (66.6)	168.3 (73.4)	142.3 (78.9)	158.6 (70.3)	161.6 (58.9)	162.3 (68.1)	133.3 (35.4)	150.1 (58.4)	129.5 (82.0)	155.1 (51.0)	140.6 (45.7)	152.7 (75.2)	116.2 (61.5)
**Cereals/Grains (g/day)**	187.5 (51.3)	191.0 (150.4)	218.6 (93.5)	124.0 (25.7)	149.2 (36.4)	141.5 (47.4)	176.3 (54.6)	166.4 (59.8)	139.2 (51.1)	157.0 (57.9)	196.8 (64.3)	203.7 (71.3)	164.0 (63.1)	145.3 (46.9)	170.1 (35.5)	174.9 (42.4)	160.0 (62.3)	171.9 (60.8)
**Fruits (g/day)**	126.6 (100.7)	30.0 (42.4)	124.4 (116.1)	44.7 (61.4)	99.8 (91.4)	139.3 (124.9)	97.0 (83.8)	72.5 (79.7)	135.4 (107.3)	197.0 (165.4)	69.1 (58.7)	160.4 (175.9)	100.8 (124.3)	105.7 (92.9)	146.0 (110.5)	145.5 (73.5)	69.0 (93.9)	87.5 (79.1)
**Eggs (g/day)**	29.1 (19.5)	2.7 (3.8)	24.0 (34.0)	28.6 (34.5)	22.6 (23.7)	30.4 (20.6)	28.8 (31.4)	21.0 (19.6)	25.3 (23.5)	24.8 (13.1)	19.3 (23.6)	21.3 (23.0)	31.5 (37.4)	36.2 (42.6)	39.0 (18.1)	34.1 (18.1)	29.3 (22.4)	23.5 (20.8)
**Milk and dairy products (g/day)**	320.2 (95.5)	232.5 (100.2)	480.0 (127.8)	171.0 (161.8)	272.5 (129.7)	300.4 (99.0)	432.3 (147.5)	305.8 (136.3)	391.5 (119.8)	357.2 (96.5)	421.9 (176.3)	322.4 (180.2)	443.6 (201.8)	282.8 (102.4)	415.7 (109.4)	364.8 (145.3)	337.4 (131.3)	374.4 (187.7)
**Pulses (g/day)**	4.2 (10.2)	16.7 (9.4)	9.5 (11.3)	16.0 (13.6)	16.5 (19.0)	14.8 (20.3)	6.2 (9.3)	8.1 (10.4)	16.0 (13.5)	15.3 (18.3)	6.6 (10.4)	9.8 (11.4)	7.8 (12.5)	16.4 (11.5)	26.5 (24.5)	19.8 (13.4)	10.5 (13.8)	12.6 (10.8)
**Fish and shellfish (g/day)**	26.2 (32.8)	35.3 (23.6)	39.0 (49.5)	13.5 (18.6)	48.3 (49.0)	59.0 (59.0)	65.8 (64.5)	39.4 (41.8)	22.2 (38.8)	60.8 (41.5)	23.9 (30.5)	21.1 (24.7)	48.5 (46.2)	60.0 (62.8)	71.2 (130.5)	43.0 (45.5)	47.0 (49.1)	53.2 (56.9)
**Ready-to-eat meals (g/day)**	143.8 (131.2)	20.0 (28.3)	96.9 (96.0)	77.0 (39.2)	80.4 (61.1)	65.6 (73.5)	89.7 (65.8)	102.6 (96.8)	106.5 (72.2)	28.1 (28.0)	99.8 (74.4)	104.8 (128.3)	58.8 (35.1)	55.2 (36.3)	77.4 (61.3)	65.7 (69.2)	80.6 (73.1)	81.8 (68.9)
**Sauces and condiments (g/day)**	13.1 (9.2)	1.8 (2.5)	9.4 (5.3)	10.1 (11.4)	10.3 (9.8)	7.2 (7.5)	14.2 (13.2	22.9 (13.8)	16.4 (17.4)	9.3 (8.1)	12.4 (9.2)	15.1 (11.0)	23.3 (18.2)	18.9 (27.8)	8.1 (7.1)	28.0 (31.7)	16.6 (13.6)	21.2 (27.9)
**Supplements and meal replacements (g/day)**	0.0 (0.0)	0.0 (0.0)	0.0 (0.0)	0.0 (0.0)	0.0 (0.0)	0.0 (0.0)	0.0 (0.0)	0.0 (0.0)	0.0 (0.0)	0.0 (0.0)	0.0 (0.0)	0.0 (0.0)	0.0 (0.0)	0.0 (0.0)	0.0 (0.0)	0.0 (0.0)	0.1 (0.5)	0.0 (0.0)
**Vegetables (g/day)**	97.7 (51.6)	156.7 (14.1)	154.6 (63.5)	120.9 (76.9)	123.2 (100.0)	142.2 (137.6)	117.4 (71.8	112.9 (82.7)	121.5 (101.7)	205.1 (213.9)	141.7 (87.2)	168.7 (84.7)	96.6 (37.8)	132.9 (58.4)	155.7 (109.3)	122.5 (112.4)	106.3 (64.8)	86.1 (67.9)

Data reported as means (standard error of the mean, SEM) per group. * p<0.05 difference Northwest (male) (Games-Howell test) ** p<0.01 difference Central (male) (Games Howell test).

Table S3. Intake of major food groups (g/day) from the ANIBES Study amongst children (boys and girls, 9 to 12 years) assessed by habitat size: rural, semi-urban and urban.

	Rural		Semi-urban		Urban	
	Boys	*Girls*	*Boys*	*Girls*	*Boys*	*Girls*
**n**	**46**	**33**	**50**	**30**	**30**	**24**
**Oils and Fats (g/day)**	22.9 (10.2)	22.8 (9.2)	24.8 (10.1)	24.8 (9.0)	20.8 (9.0)	20.7 (7.9)
**Appetizers (g/day)**	5.7 (9.9)	4.8 (8.7)	5.7 (11.3)	6.1 (12.0)	3.0 (6.3)	4.5 (11.8)
**Sugars and sweets (g/day)**	28.6 (24.0)	25.9 (21.0)	23.5 (20.5)	25.2 (17.8)	19.3 (15.4)	22.9 (15.1)
**Alcoholic beverages (g/day)**	0.0 (0.0)	0.0 (0.0)	0.0 (0.0)	0.0 (0.0)	0.0 (0.0)	0.0 (0.0)
**Non-alcoholic beverages (g/day)**	709.7 (417.6	650.8 (381.6)	710.8 (476.8)	684.7 (389.3)	786.3 (396.2)	706.6 (331.1
**Meat and meat products (g/day)**	135.0 (51.9)	132.0 (60.3	171.1* (85.8)	143.6 (64.5)	177.1* (69.7)	119.5 (57.0)
**Cereals/Grains (g/day)**	165.8 (60.0)	153.6 (51.8)	173.6 (57.2)	180.7 (49.3)	170.0 (66.6)	165.9 (74.2)
**Fruits (g/day)**	100.2 (95.6)	144.2 (133.0)	90.4 (100.6)	92.3 (67.9)	101.6 (91.9)	110.3 (127.9)
**Eggs (g/day)**	28.2 (27.9)	31.3 (26.2)	30.0 (26.6)	26.4 (19.4)	23.2 (19.9)	18.5 (19.0)
**Milk and dairy products (g/day)**	342.7 (127.2)	345.4 (157.7)	418.1* (169.8)	280.5 (123.1)	391.4 (129.4)	353.1 (165.9)
**Pulses (g/day)**	8.0 (12.3)	12.3 (12.2)	10.6 (17.0)	15.1 (14.0)	16.3 (13.2)	14.2 (14.8)
**Fish and shellfish (g/day)**	50.4 (67.8)	49.6 (55.0)	46.7 (53.9)	38.0 (45.3)	36.9 (55.2)	46.8 (37.9)
**Ready-to-eat meals (g/day)**	75.3 (67.5)	74.0 (69.6)	87.9 (71.5)	72.8 (88.5)	111.6 (79.8)	77.2 (79.2)
**Sauces and condiments (g/day)**	13.9 (13.7)	16.8 (16.7)	14.7 (12.4)	18.5 (24.6)	14.6 (12.8)	18.8 (23.9)
**Supplements and meal replacements (g/day)**	0.0 (0.0)	0.0 (0.0)	0.1 (0.4)	0.0 (0.0)	0.0 (0.0)	0.0 (0.0)
**Vegetables (g/day)**	103.7 (61.2)	133.2 (107.2)	127.5 (81.2)	115.8 (66.9)	135.2 (92.1)	145.9 (155.1)

Data reported as means (standard error of the mean, SEM) per group. * $p<0.05$ difference rural (Games-Howell test).

Table S4. Intake of major food groups (g/day) from the ANIBES Study amongst adolescents (male/female, 13 to 17 years) segmented by geographical areas (Nielsen areas).

	Barcelona (Metropolitan Area)		Canary Islands		Center		East		Madrid (Metropolitan Area)		Northeast		Northwest		North Central		South	
	Male	*Female*	*Male*	*Female*	*Male*	*Female*	*Male*	*Female*	*Male*	*Female*	*Male*	*Female*	*Male*	*Female*	*Male*	*Female*	*Male*	*Female*
**n**	5	2	8	5	15	11	32	10	10	7	15	3	19	11	12	6	21	19
**Oils and Fats (g/day)**	23.6 (12.4)	26.8 (22.4)	26.4 (17.0)	15.6 (7.1)	24.6 (12.7)	18.4 (10.4)	20.8 (9.2)	16.0 (6.0)	19.8 (14.1)	16.1 (8.0)	24.2 (10.0)	24.1 (16.1)	22.3 (17.5)	23.8 (7.6)	25.2 (15.6)	22.6 (12.4)	23.7 (11.2)	24.4 (15.7)
**Appetizers (g/day)**	6.0 (8.9)	1.7 (2.4)	7.7 (19.3)	1.0 (2.2)	4.0 (11.8)	1.5 (5.3)	7.5 (17.4)	2.3 (3.5	4.3 (8.3)	9.7 (14.5)	7.8 (11.7)	0.0 (0.0)	0.9 (2.4)	4.8 (8.3)	6.8 (11.5)	16.1 (19.6)	4.1 (9.3)	10.3 (18.5)
**Sugars and sweets (g/day)**	15.2 (7.5)	17.2 (9.2)	23.7 (19.7)	6.5 (2.9)	25.5 (21.9)	28.6 (18.1)	23.1 (16.1)	34.0 (39.6)	17.0 (11.7)	16.5 (10.0)	15.3 (13.5)	22.8 (2.0)	17.1 (13.0)	24.9 (18.4)	22.2 (21.8)	33.9 (36.9)	22.2 (16.0)	20.9 (15.4)
**Alcoholic beverages (g/day)**	0.0 (0.0)	0.0 (0.0)	0.0 (0.0)	0.0 (0.0)	0.0 (0.0)	15.2 (50.3)	0.0 (0.0)	5.0 (15.8)	0.0 (0.0)	0.0 (0.0)	0.0 (0.0)	0.0 (0.0	0.0 (0.0)	0.0 (0.0)	0.0 (0.0)	0.0 (0.0)	7.4 (33.8)	0.0 (0.0)
**Non-alcoholic beverages (g/day)**	508.7 (435.2)	698.3 (101.4)	646.9 (471.4)	575.3 (293.9)	608.3 (302.8)	691.7 (210.0)	849.1 (495.8)	772.2 (421.5)	758.7 (283.8)	724.43 (475.7)	607.6 (284.2)	571.1 (101.8)	719.6 (519.8)	531.3 (284.8)	788.8 (443.9)	565.3 (169.4)	664.8 (346.0)	743.6 (271.3)
**Meat and meat products (g/day)**	198.1 (120.8)	169.2 (53.0)	144.0 (55.2)	106.0 (70.6)	184.9 (87.8)	134.6 (66.2)	157.1 (66.4)	144.1 (69.2)	190.7 (89.0)	116.3 (53.7)	199.8 (56.3)	101.7 (54.1)	156.0 (66.9)	148.7 (81.6)	178.2 (66.9)	134.0 (80.1)	167.8 (64.5)	141.6 (62.4)
**Cereals/Grains (g/day)**	170.3 (56.9)	204.2 (20.0)	181.1 (33.6)	147.1 (69.5)	202.2 (69.8)	148.5 (58.9)	206.7 (73.5)	190.3 (80.0)	174.0 (104.4)	105.2 (56.4)	228.0 (72.2)	101.7 (43.1)	176.3 (63.7)	191.3 (69.1)	174.2 (75.9)	173.1 (45.8)	183.1 (86.1)	152.3 (46.3)
**Fruits (g/day)**	12.8 (24.7)	244.7 (180.0)	158.2 (152.5)	96.0 (85.5)	107.7 (165.1)	111.9 (109.5)	63.0 (74.2)	143.6 (209.7)	52.3 (75.8)	61.9 (69.3)	79.2 (128.6)	74.6 (70.4)	116.6 (156.3)	84.4 (77.5)	104.5 (134.0)	67.8 (85.8)	52.0 (60.6)	78.4 (69.5)
**Eggs (g/day)**	33.2 (10.9)	1.7 (2.4)	14.0 (19.1)	25.6 (46.3)	35.3 (19.4)	16.3 (10.9)	37.2 (40.1)	11.4 (18.3)	28.9 (28.9)	13.6 (12.0)	39.9 (57.3)	18.7 (4.6)	34.2 (30.6)	36.4 (27.3)	47.1 (44.1)	34.9 (42.7)	31.4 (32.3)	35.1 (36.6)
**Milk and dairy products (g/day)**	286.0 (108.9)	295.8 (8.3)	457.5 (267.9)	246.3 (230.4)	375.9 (235.9)	215.9 (162.2)	321.3 (210.4)	212.6 (186.0)	273.5 (207.1)	220.2 (124.2)	291.2 (114.3)	328.2 (198.9)	362.1 (175.0)	297.0 (99.6)	235.9 (130.8)	319.5 (163.5)	362.4 (229.5)	279.6 (157.2)
**Pulses (g/day)**	6.7 (14.9)	13.3 (18.9)	11.0 (18.8)	12.0 (14.5)	21.7 (17.5)	16.1 (23.8)	6.7 (10.5)	13.4 (15.8)	15.3 (20.9)	5.7 (7.1)	6.7 (11.1)	23.3 (6.7)	12.5 (16.5)	14.3 (27.6)	14.4 (15.9)	11.1 (17.7)	15.4 (14.7)	8.9 (13.0)
**Fish and shellfish (g/day)**	26.7 (20.6)	98.7 (139.5)	23.0 (25.9)	44.8 (33.2)	42.1 (42.4)	16.2 (22.9)	55.1 (68.5)	12.2 (28.2)	41.1 (51.1)	95.5 (97.4)	31.3 (40.5)	67.8 (74.9)	46.0 (86.2)	50.3 (88.1)	29.8 (29.7)	8.7 (14.1)	32.2 (41.5)	40.9 (54.2)
**Ready-to-eat meals (g/day)**	81.8 (56.9)	0.0 (0.0)	95.5 (87.4)	84.0 (117.0)	107.0 (82.5)	69.7 (64.6)	121.7 (110.9)	74.2 (83.7)	109.6 (79.6)	114.3 (57.2)	119.3 (146.7)	102.5 (99.7)	103.4 (130.6)	62.4 (67.7)	104.2 (54.4)	82.8 (75.3)	92.9 (81.9)	66.6 (67.7)
**Sauces and condiments (g/day)**	41.4 (25.6)	5.3 (3.9)	14.3 (12.2)	12.3 (12.7)	13.2 (13.3)	12.6 (11.1)	20. 5 (15.4)	23.5 (37.0)	12.0 (6.1)	11.2 (9.7)	15.2 (11.8)	5.2 (5.0)	10.1 (9.2)	13.8 (11.5)	13.7 (11. 6)	13.0 (10.4)	15.3 (11.4)	15.3 (12.2)
**Supplements and meal replacements (g/day)**	0.00 (0.0)	0.00 (0.0)	2.5 (7.1)	0.00 (0.0)	0.00 (0.0)	0.00 (0.0)	0.00 (0.0)	0.00 (0.0)	0.00 (0.0)	0.00 (0.0)	0.7 (2.6)	0.00 (0.0)	0.00 (0.0)	0.00 (0.0)	0.00 (0.0)	0.00 (0.0)	0.00 (0.0)	0.00 (0.0)
**Vegetables (g/day)**	133.1 (98.9)	243.8 (71.0)	140.6 (89.8)	124.0 (91.9)	136.0 (104.6)	103.0 (54.6)	102.7 (78.5)	98.4 (48.6)	113.4 (105.2)	120.0 (51.1)	115.0 (55.4)	184.4 (131.3)	122.2 (78.7)	133.5 (80.9)	156.9 (138.7)	113.4 (64.5)	131.6 (89.3)	109.4 (86.1)

Data reported as means (standard error of the mean, SEM) per group.

Table S5. Intake of major food groups (g/day) from the ANIBES Study amongst adolescents (male/female, 13 to 17 years) assessed by habitat size: rural, semi-urban and urban.

	Rural		Semi-urban		Urban	
	Male	*Female*	*Male*	*Female*	*Male*	*Female*
**n**	58	23	46	33	33	18
**Oils and Fats (g/day)**	22.9 (11.7)	19.8 (13.6)	22.7 (14.6)	21.3 (11.7)	23.5 (11.7)	21.2 (8.9)
**Appetizers (g/day)**	6.5 (12.9)	9.0 (16.8)	4.1 (10.0)	4.9 (10.5)	5.2 (14.4)	5.0 (11.1)
**Sugars and sweets (g/day)**	24.4 (18.6)	25.8 (17.1)	19.2 (14.6)	27.3 (28.4)	16.6 (13.5)	15.8 (13.0)
**Alcoholic beverages (g/day)**	0.0 (0.0)	0.0 (0.0)	3.4 (22.9)	5.1 (29.0)	0.0 (0.0)	2.8 (11.8)
**Non-alcoholic beverages (g/day)**	647.4 (339.5)	626.9 (239.4)	807.0 (461.5)	652.8 (263.0)	701.2 (467.6)	766.2 (416.4)
**Meat and meat products (g/day)**	170.7 (75.6)	144.3 (66.9)	164.2 (63.3)	126.1 (66.5)	182.5 (75.1)	142.2 (65.1)
**Cereals/Grains (g/day)**	203.1* (58.2)	182.8 (68.4)	202.3 (85.5)	150.0 (56.7)	160.8 (77.1)	144.6 (62.3)
**Fruits (g/day)**	102.6 (149.6)	92.6 (96.9)	69.1 (81.6)	94.2 (102.1)	63.8 (86.7)	104.3 (143.6)
**Eggs (g/day)**	36.0 (40.5)	31.4 (36.6)	29.5 (24.5)	22.4 (25.4)	39.7 (41.2)	21.7 (26.0)
**Milk and dairy products (g/day)**	365.1 (221.3)	270.7 (149.8)	331.4 (176.3)	265.9 (150.0)	273.1 (183.1)	241.5 (176.4)
**Pulses (g/day)**	11.5 (14.8)	8.8 (14.7)	12.5 (15.0)	13.9 (16.4)	12.3 (17.0)	13.4 (23.1)
**Fish and shellfish (g/day)**	39.8 (45.5)	36.3 (47.7)	48.4 (74.4)	36.6 (63.5)	29.2 (36.4)	51.9 (78.7)
**Ready-to-eat meals (g/day)**	102.4** (104.6)	43.2** (44.9)	113.9 (106.2)	97.8 (82.4)	107.4 (89.8)	70.3 (69.3)
**Sauces and condiments (g/day)**	14.3 (12.3)	13.3 (10.8)	17.4 (12.8)	11.8 (11.3)	17.2 (17.7)	20.2 (27.9)
**Supplements and meal replacements (g/day)**	0.3 (2.6)	0.0 (0.0)	0.2 (1.5)	0.0 (0.0)	0.0 (0.0)	0.0 (0.0)
**Vegetables (g/day)**	128.5 (100.0)	121.6 (84.9)	108.4 (76.5)	114.1 (63.4)	136.5 (88.3)	126.7 (86.0)

Data reported as means (standard error of the mean, SEM) per group.* $p<0.05$ difference urban (Games-Howell test); ** $p<0.01$ difference semi-urban (Games-Howell test).

Table S6. Intake of major food groups (g/day) from the ANIBES Study amongst adult population (male/female, 18 to 64 years) segmented by geographical areas (Nielsen areas)

	Barcelona (Metropolitan Area)		Canary Islands		Center		East		Madrid (Metropolitan Area)		Northeast		Northwest		North Central		South	
	Male	*Female*	*Male*	*Female*	*Male*	*Female*	*Male*	*Female*	*Male*	*Female*	*Male*	*Female*	*Male*	*Female*	*Male*	*Female*	*Male*	*Female*
**n**	46	63	32	42	89	73	132	139	108	109	88	102	73	67	64	77	166	185
**Oils and Fats (g/day)**	23.4 (9.6)	25.5 (11.7)	22.7 (10.0)	19.8×× (8.9)	26.4 (12.4)	22.1×× (11.0)	24.1 (10.9)	23.4×× (9.1)	22.4 (9.3)	23.3×× (11.4)	26.1 (12.1)	23.8 (11.2)	23.3 (11.3)	21.1×× (8.5)	27.4 (11.4)	26.9 (10.1)	25.7 (12.1)	28.1 (11.8)
**Appetizers (g/day)**	6.1 (10.4)	5.1 (11.5)	6.5 (11.6)	3.0• (5.8)	6.4 (17.3)	4.5 (13.2)	9.3 (13.4)	7.9 (15.0)	2.8*** (6.6)	2.1•• (6.5)	6.8 (12.3)	6.4 (14.8)	3.3*** (7.4)	3.9 (8.1)	6.8 (13.8)	6.9 (12.7)	7.5* (16.4)	5.4 (11.5)
**Sugars and sweets (g/day)**	12.2 (12.3)	13.8 (13.4)	17.1 (14.0)	17.9 (16.1)	16.9 (16.4)	19.3 (19.6)	12.8 (15.4)	16.0 (14.3)	16.0 (13.9)	14.9 (14.3)	14.8 (13.8)	16.5 (15.7)	20.2^ (19.7)	18.2 (15.6)	18.9 (19.6)	20.3 (17.7)	11.0 (12.9)	14.4 (14.0)
**Alcoholic beverages (g/day)**	102.5 (167.7)	26.8 (51.3)	202.9 (331.7)	66.5 (123.0)	124.7 (191.1)	101.8◊◊ (160.1)	195.1 (238.4)	99.8◊◊ (165.6)	129.9 (213.7)	53.3 (156.9)	117.1 (257.5)	74.1 (157.0)	140.2 (276.2)	53.8 (89.8)	148.6 (222.1)	58.4 (126.7)	210.6 (312.2)	71.9◊◊ (138.9)
**Non-alcoholic beverages (g/day)**	851.3 (480.4)	850.4 (519.3)	798.8 (431.7)	929.7 (491.7)	819.3 (481.4)	844.4 (435.0)	1010.2 (674.8)	1049.8 (537.8)	930.1 (528.8)	874.0 (470.8)	920.4 (545.4)	935.5 (597.1)	778.5 (587.2)	720.4▫▫▫ (416.5)	997.8 (526.1)	948.2 (585.1)	798.0 (555.6)	795.3▫▫▫ (452.5)
**Meat and meat products (g/day)**	166.9 (78.6)	137.0 (85.6)	143.3 (72.5)	98.2 (61.0)	160.2 (89.0)	112.3 (70.3)	178.7 (89.6)	128.9 (68.6)	183.3 (96.7)	137.6 (71.7)	179.7 (98.8)	125.6 (76.9)	142.6 (77.8)	133.5 (83.8)	197.5▪▪ (93.6)	138.8 (79.2)	158.1 (87.6)	131.3 (68.1)
**Cereals/Grains (g/day)**	170.6 (81.4)	132.1 (53.7)	159.0 (56.9)	135.0 (52.3	169.0 (69.2)	141.7 (61.2)	170.4 (68.4)	145.3 (57.8)	167.9 (72.5)	121.8 (53.4)	168.5 (72.5)	135.4 (54.9)	151.5 (69.6)	124.3 (59.5)	163.6 (66.3)	136.5 (62.7)	154.8 (61.6)	136.0 (52.9)
**Fruits (g/day)**	168.3 (159.0	191.6 (165.3)	221.0 (253.3)	149.3 (165.2)	131.9 (143.6)	138.8 (148.1)	167.4 (165.5)	172.7 (170.8)	141.0 (180.9)	174.0 (179.7)	141.1 (167.9)	120.0 (157.1)	144.5 (174.9)	184.8 (202.3)	175.4 (184.2	179.0 (241.2)	118.4 (138.2)	125.6 (136.9)
**Eggs (g/day)**	23.8 (26.8)	23.4 (25.3)	28.1 (27.1)	20.5 (20.6)	40.0# (33.2)	30.3 (26.5)	26.4 (28.4)	27.2 (26.0)	33.1 (30.0)	26.4 (21.2)	34.6 (46.8)	23.5 (21.7)	38.6 (35.2)	28.7 (26.5)	41.4 (34.5)	30.5 (28.8)	32.5 (31.0)	22.1 (24.9)
**Milk and dairy products (g/day)**	232.1 (171.4)	232.4 (128.9)	280.3 (200.5)	266.1 (172.7)	265.4 (145.8)	273.0 (146.9)	212.9+ (183.4)	250.1 (134.7)	283.4 (158.4)	252.4 (137.4)	234.3 (201.7)	221.7 (133.3)	262.2 (160.9)	260.7 (166.4)	273.7 (153.9)	292.2 (166.4)	225.4 (141.0)	229.5 (127.7)
**Pulses (g/day)**	11.5 (14.9)	16.0 (20.9)	15.3 (19.0)	11.3 (17.5)	14.9 (21.1)	11.0 (13.9)	10.7 (15.0)	11.7 (14.4)	15.0 (23.1)	12.0 (16.7)	16.7 (23.5)	10.7 (15.8)	13.1 (16.2)	14.0 (21.8)	17.4 (20.2)	12.0 (16.6)	15.0 (17.6)	13.9 (16.3)
**Fish and shellfish (g/day)**	82.1 (91.0)	90.0 (91.1)	51.7 (67.4)	45.6 (51.0)	62.0 (58.5)	65.6 (101.3)	65.7 (69.3)	61.0 (66.2)	52.9 (55.9)	65.8 (71.4)	48.4 (63.9)	48.7 (61.2)	67.5 (72.8)	67.2 (61.0)	77.9 (85.7)	64.8 (64.3)	57.2 (65.1)	65.5 (70.5)
**Ready-to-eat meals (g/day)**	84.9 (87.9)	50.2 (71.1)	67.0 (93.6)	50.5 (54.3)	75.5 (94.9)	71.7 (74.2)	67.7 (79.7)	54.8 (58.7)	89.1 (89.2)	55.8 (61.0)	86.3 (94.2)	79.4 (74.4)	59.2 (78.9)	52.6 (58.2)	52.0 (69.3)	52.7 (52.7)	75.5 (74.9)	68.8 (73.4)
**Sauces and condiments (g/day)**	13.6 (15.6)	10.5 (10.4)	12.6 (12.3)	13.8 (11.2)	14.9 (15.4)	13.7 (16.1)	17.0 (17.6)	12.9 (13.0)	13.8 (12.9)	9.1 (12.2)	16.9 (20.4)	10.9 (12.9)	11.9 (12.1)	9.6 (13.2)	14.5 (15.4)	12.6 (14.0)	15.4 (16.2)	12.9 (14.6)
**Supplements and meal replacements (g/day)**	0.1 (1.0)	0.5 (2.1)	1.7 (6.3)	0.3 (1.5)	0.6 (3.6)	0.1 (1.2)	0.0 (0.2)	0.3 (1.7)	0.0 (0.1)	0.0 (0.0)	1.9 (15.3)	1.4 (11.7)	0.2 (1.2)	0.1 (0.8)	0.1 (0.9)	0.6 (3.5)	0.6 (4.1)	0.0 (0.0)
**Vegetables (g/day)**	169.4 (90.4)	208.8 (128.2)	209.6 (114.4)	179.5 (113.5)	180.4 (120.6)	158.1 (108.3)	184.7 (123.8)	185.9 (112.3)	170.2 (103.3)	177.6 (108.4)	206.9 (143.1)	194.8 (125.8)	182.2 (109.8)	170.6 (97.3)	202.6 (141.6)	204.0 (132.8)	160.7 (107.7)	166.9 (93.5)

Data reported as means (standard error of the mean, SEM) per group. * p<0.05 difference (Metropolitan Area) Madrid (male) (Games-Howell test); *** p<0.001 difference East (male) (Games-Howell test); ^ p<0.05 difference South (male) (Games-Howell test); ▪▪ p<0.01 difference Northwest (male) (Games-Howell test); #p<0.05 difference East (male) (Games-Howell test); + p<0.05 difference (Metropolitan Area) Madrid (male) (Games-Howell test); ××p<0.01 difference South (female) (Games-Howell test); • p<0.05 difference East (female) (Games-Howell test); •• p<0.01 difference East (female) (Games-Howell test); ◊◊ p<0.01 difference Barcelona (Metropolitan Area (female) (Games-Howell test); ▫▫▫ p<0.01 difference East (female) (Games-Howell test).

Table S7. Intake of major food groups (g/day) from the ANIBES Study amongst adults (male/female, 18 to 64 years) assessed by habitat size: rural, semi-urban and urban.

	Rural		Semi-urban		Urban	
	Male	*Female*	*Male*	*Female*	*Male*	*Female*
**n**	266	298	284	277	248	282
**Oils and Fats (g/day)**	25.5** (11.0)	25.0 (11.8)	26.0** (11.8)	24.1 (9.7)	22.6 (10.7)	24.3 (11.1)
**Appetizers (g/day)**	8.2* (14.5)	5.4 (11.8)	6.1 (13.0)	5.8 (12.7)	4.9 (12.0)	4.7 (11.6)
**Sugars and sweets (g/day)**	13.4 (14.4)	17.9 (16.4)	16.6 (16.9)	15.7 (15.8)	14.4 (14.9)	15.4 (13.9)
**Alcoholic beverages (g/day)**	189.6× (293.3)	81.1 (156.1)	134.4 (212.1)	59.8 (113.2)	155.2 253.9)	70.2 (150.9)
**Non-alcoholic beverages (g/day)**	829.6 (546.4)	876.0 (563.4)	935.7 (552.3)	891.7 (485.4)	883.8 (583.8)	891.0 (476.7)
**Meat and meat products (g/day)**	169.6 (93.7)	123.8 (72.9)	168.9 (83.3)	129.2 (73.0)	169.1 (94.3)	134.5 (74.9)
**Cereals/Grains (g/day)**	164.1 (64.1)	137.1 (54.5)	168.3 (72.4)	135.5 (56.3	158.1 (68.5)	132.0 (58.6)
**Fruits (g/day)**	150.3 (171.3)	147.1 (164.8)	157.8 (166.0)	147.6 (166.0)	132.7 (165.7)	171.6 (186.4)
**Eggs (g/day)**	33.6 (37.6)	24.9 (25.4)	34.4 (31.4)	25.1 (23.5)	31.5 (30.7)	26.9 (25.6)
**Milk and dairy products (g/day)**	221.5## (168.0)	243.5 (143.1)	263.6 (154.3)	260.8 (145.9)	253.6 (177.1)	242.0 (139.0)
**Pulses (g/day)**	15.7+ (19.4)	12.7 (16.6)	15.2 (20.8)	12.9 (16.2)	11.8 (16.9)	12.0 (17.5)
**Fish and shellfish (g/day)**	59.5 (68.1)	62.6 (65.6)	67.1 (72.6)	59.1 (60.4)	56.9 (64.0)	69.4 (87.5)
**Ready-to-eat meals (g/day)**	67.3 (77.9)	63.3 (65.6)	75.2 (86.7)	63.1 (66.3)	80.0 (87.3)	57.8 (67.6)
**Sauces and condiments (g/day)**	14.1 (16.8)	11.6 (13.7)	14.8 (14.7)	13.1 (13.6)	16.1 (16.3)	10.8 (12.9)
**Supplements and meal replacements (g/day)**	0.8 (9.1)	0.1 (0.8)	0.7 (3.8)	0.4 (2.4)	0.1^ (0.8)	0.6 (7.1)
**Vegetables (g/day)**	185.4 (125.6)	177.3 (107.3)	182.8 (124.2)	177.8 (113.2)	174.3 (104.0)	188.8 (116.1)

Data reported as means (standard error of the mean, SEM) per group. ** p<0.01 difference Urban (Games-Howell test); *p<0.05 difference Urban (Games-Howell test); × p<0.05 difference semi-urban (Games-Howell test); ## p<0.01 difference semi-urban (Games-Howell test); + p<0.05 difference Urban (Games-Howell test); ^ p<0.05 difference Urban (Games-Howell test).

Table S8. Intake of major food groups (g/day) from the ANIBES Study amongst senior population (male/female, 65 to 75 years) segmented by geographical areas (Nielsen areas).

	Barcelona (Metropolitan Area)		Canary Islands		Center		East		Madrid (Metropolitan Area)		Northeast		Northwest		North Central		South	
	Male	*Female*	*Male*	*Female*	*Male*	*Female*	*Male*	*Female*	*Male*	*Female*	*Male*	*Female*	*Male*	*Female*	*Male*	*Female*	*Male*	*Female*
**n**	2	6	7	4	7	5	12	14	11	16	9	11	11	9	12	10	28	32
**Oils and Fats (g/day)**	39.6 (15.0)	29.4 (6.5)	31.8 (25.2)	14.8 (4.7)	22.9 (7.1)	21.4 (7.6)	23.6 (6.5)	22.2 (11.8)	24.4 (6.6)	20.5 (10.5)	29.1 (13.3)	26.6 (11.6)	24.9 (8.8)	19.1 (7.7)	30.2 (8.7)	39.1 (22.8)	29.7 (16.9)	29.1× (11.9)
**Appetizers (g/day)**	3.3 (4.7)	15.4 (35.1)	2.6 (4.1)	0.3 (0.7)	0.8 (1.4)	0.0 (0.0)	1.7 (3.2)	2.6 (5.5)	2.4 (5.4)	1.4 (4.1)	1.9 (5.8)	5.5 (10.5)	7.5 (17.0)	0.0 (0.0)	1.7 (2.6)	2.0 (2.8)	4.6 (10.0)	1.5 (4.2)
**Sugars and sweets (g/day)**	8.5 (12.0)	8.6 (8.2)	13.0 (20.0)	6.7 (8.3)	8.1 (12.1)	24.0 (16.7)	10.4 (10.8)	16.5 (14.6)	16.6 (18.5)	7.9 (9.3)	14.7 (16.5)	10.6 (12.5)	11.6 (11.6)	9.6 (9.8)	17.6 (20.6)	28.5 (18.1)	11.3 (8.7)	7.3 (10.0)
**Alcoholic beverages (g/day)**	339.2 (147.3)	56.7 (78.3)	23.8 (49.9)	41.7 (83.3)	96.7 (192.6)	20.0 (44.7)	126.1 (181.2)	33.6 (77.9)	103.3 (156.8)	21.0 (45.3)	41.1 (73.3)	59.8 (66.7)	184.8 (169.6)	24.7 (38.0)	181.4 (212.3)	147.3 (232.8)	194.6 (227.1)	58.9 (89.0)
**Non-alcoholic beverages (g/day)**	652.5 (203.9)	865.0 (250.4)	740.9 (147.2)	752.2 (227.9)	733.1 (754.7)	1318.5 (526.3)	770.8 (353.1)	873.3 (313.9)	596.5 (257.2)	740.8 (374.1)	748.3 (523.2)	1008.5 (669.8)	289.9 (195.6)	413.6 (303.9)	846.0 (518.7)	860.3 (537.6)	569.0 (406.2)	580.3 (289.2)
**Meat and meat products (g/day)**	205.8 (24.7)	79.0 (65.5)	146.7 (129.7)	47.1 (38.0)	127.6 (88.2)	90.3 (46.7)	112.9 (83.7)	82.4 (52.9)	167.1 (62.1)	113.8 (64.4)	156.5 (50.4)	123.8 (84.2)	112.0 (54.1)	110.4 (36.6)	150.8 (58.7)	96.6 (44.9)	112.2 (76.9)	111.9 (63.9)
**Cereals/Grains (g/day)**	221.8 (32.8)	111.8 (50.7)	191.6 (54.8)	130.9 (64.9)	127.4 (45.8)	88.2 (26.8)	139.7 (95.6)	114.9 (46.5)	113.8 (37.9)	116.8 (35.1)	155.9 (58.9)	89.4 (46.4)	105.0 (49.5)	113.0 (43.3)	133.5 (53.1)	139.1 (56.0)	136.4 (45.1)	122.7 (44.1)
**Fruits (g/day)**	205.0 (77.8)	255.2 (72.1)	261.5 (206.4)	451.8 (159.2)	422.1 (239.3)	215.2 (114.9)	337.3 (246.8)	349.1 (156.8)	360.4 (271.7)	262.0 (165.8)	365.8 (270.1)	242.9 (138.3)	120.1 (103.7)	213.6 (155.4)	434.8 (314.4)	458.4 (347.2)	163.6 (172.5)	236.1 (198.2)
**Eggs (g/day)**	73.7 (58.9)	59.4 (41.5)	49.0 (24.7)	10.8 (11.5)	25.9 (18.5)	26.3 (18.0)	29.1 (21.8)	11.5 (13.6)	23.7 (33.8)	20.1 (21.9)	15.2 (20.2)	29.7 (22.8)	39.4 (35.0)	46.1 (34.7)	62.1** (29.3)	40.6 (42.1)	29.3 (23.0)	34.0# (26.0)
**Milk and dairy products (g/day)**	0.0 (0.0)	173.4 (46.7)	289.0 (171.4)	363.3 (279.5)	285.4 (83.3)	266.2 (103.5)	247.9 (174.5)	263.9 (181.6)	243.1 (168.8)	399.4•• (170.8)	241.2 (100.4)	215.8 (129.3)	202.6 (162.5)	271.1 (135.2)	345.3 (215.6)	258.5 (187.1)	206.3 (132.7)	261.1 (124.4)
**Pulses (g/day)**	11.7 (16.5)	18.6 (15.6)	17.9 (21.4)	29.6 (25.3)	17.1 (24.1)	18.0 (10.7)	14.0 (18.0)	19.4 (30.3)	9.7 (10.1)	12.0 (11.6)	20.6 (22.8)	10.3 (20.9)	13.3 (14.6)	12.6 (9.7)	13.5 (23.5)	8.5 (12.7)	24.2 (22.1)	10.2 (11.3)
**Fish and shellfish (g/day)**	86.5 (1.6)	72.2 (49.3)	41.4 (66.2)	44.8 (76.5)	120.2 (103.1)	39.3 (16.9)	80.5 (53.7)	48.0 (44.7)	105.9 (88.0)	72.6 (59.5)	132.3 (100.0)	111.4 (71.6)	84.5 (66.2)	54.8 (72.9)	99.5 (103.3)	98.4 (108.6)	78.3 (57.2)	75.4 (65.3)
**Ready-to-eat meals (g/day)**	41.7 (58.9)	38.9 (37.5)	114.3 (137.7)	39.6 (55.0)	98.8 (101.9)	11.7 (16.2)	40.8 (39.6)	46.9 (67.8)	65.2 (69.3)	38.5 (46.7)	80.9 (80.4)	35.3 (47.6)	60.3 (67.9)	54.4 (53.1)	69.4 (58.6)	60.7 (102.3)	91.1 (67.0)	52.0 (57.5)
**Sauces and condiments (g/day)**	0.5 (0.3)	1.7 (1.7)	5.1 (7.6)	6.5 (5.2)	7.4 (6.6)	1.2 (1.3)	9.3 (9.0)	3.0 (3.8)	14.0 (17.5	6.3 (8.7)	4.2 (6.4)	10.1 (11.6)	3.7 (4.2)	2.8 (2.9)	14.1 (22.0)	5.4 (6.7)	5.0 (6.7)	8.7 (11.5)
**Supplements and meal replacements (g/day)**	0.0 (0.0)	0.0 (0.0)	0.0 (0.0)	0.0 (0.0)	0.0 (0.0)	0.0 (0.0)	0.6 (1.9)	0.0 (0.0)	0.0 (0.0)	0.0 (0.0)	0.0 (0.0)	0.0 (0.0)	0.0 (0.0)	0.0 (0.0)	8.7 (23.0)	0.3 (1.1)	0.0 (0.0)	0.1 (0.5)
**Vegetables (g/day)**	319.2 (104.9)	250.3 (124.1)	178.9 (113.0)	251.5 (133.1)	197.2 (77.7)	195.9 (123.0)	241.6 (132.9)	178.5 (115.8)	211.5 (79.8)	181.4◊ (100.7)	259.3 (111.5)	254.6 (88.8)	208.3 (104.1)	228.0 (106.6)	294.9+ (100.8)	342.3 (118.7)	164.0 (92.8)	153.4◊ (79.1)

Data reported as means (standard error of the mean, SEM) per group. ** $p<0.05$ difference Northeast (Games-Howell test); + $p<0.05$ difference South (Games-Howell test). × $p<0.05$ difference Canary (Games-Howell test); # $p<0.05$ difference East (Games-Howell test); •• $p<0.01$ difference Northcentral (Games-Howell test) ◊ $p<0.05$ difference Northcentral (Games-Howell test).

Table S9. Intake of major food groups (g/day) from the ANIBES Study amongst seniors (male/female, 65 to 75 years) assessed by habitat size: rural, semi-urban and urban.

	Rural		Semi-urban		Urban	
	Male	*Female*	*Male*	*Female*	*Male*	*Female*
**n**	40	32	31	40	28	35
**Oils and Fats (g/day)**	29.7 (13.0)	26.1 (16.3)	25.1 (11.3)	26.3 (11.7)	27.7 (15.3)	25.1 (12.3)
**Appetizers (g/day)**	4.1 (11.3)	2.5 (6.9)	2.4 (4.0)	1.2 (3.3)	3.2 (7.0)	4.3 (15.0)
**Sugars and sweets (g/day)**	13.1 (13.9)	12.5 (14.4)	10.2 (12.7)	11.3 (12.8)	14.9 (15.5)	12.2 13.4)
**Alcoholic beverages (g/day)**	154.5 (192.4)	55.2 (138.4)	160.8 (221.9)	50.8 (86.7)	108.5 (140.4)	52.9 (76.0)
**Non-alcoholic beverages (g/day)**	587.5 (427.3)	860.4 (518.0)	680.0 (457.4)	670.7 (415.8)	673.7 (407.4)	757.0 (358.8)
**Meat and meat products (g/day)**	129.6 (65.1)	106.9 (42.3)	135.4 (89.4)	93.0 (58.8)	133.4 (77.2)	110.0 (75.9)
**Cereals/Grains (g/day)**	133.6 (61.7)	116.1 (45.3)	142.8 (61.9)	113.9 (51.7)	136.2 (53.2)	118.8 (38.7)
**Fruits (g/day)**	271.5 (237.0)	281.8 (157.0)	254.6 (256.3)	314.2 (253.0)	316.7 (247.5)	247.0 (160.6)
**Eggs (g/day)**	35.4 (28.8)	25.9 (20.4)	36.4 (27.7)	35.8 (31.9)	31.2 (33.2)	28.3 (31.7)
**Milk and dairy products (g/day)**	250.4 (133.6)	282.2 (157.5)	248.3 (205.0)	297.6 (144.7)	224.1 (139.7)	249.3 (173.4)
**Pulses (g/day)**	18.0** (19.3)	12.6 (20.4)	26.0** (23.5)	14.3 (16.1)	6.6 (9.7)	12.7 (14.9)
**Fish and shellfish (g/day)**	88.4 (71.6)	65.4 (74.1)	79.9 (87.7)	75.8 (68.8)	104.6 (71.2)	75.3 (61.4)
**Ready-to-eat meals (g/day)**	72.4 (69.1)	49.9 (50.3)	77.4 (74.6)	35.5 (59.8)	80.8 (83.8)	53.0 (64.1)
**Sauces and condiments (g/day)**	5.9 (8.5)	4.7 (7.1)	7.4 (13.9)	4.8 (7.2)	9.9 (12.7)	8.8 (11.2)
**Supplements and meal replacements (g/day)**	0.6 (2.7)	0.0 (0.0)	2.6 (14.4)	0.1 (0.5)	0.2 (1.3)	0.1 (0.5)
**Vegetables (g/day)**	222.0 (102.1)	213.2 (112.0)	191.7 (96.8)	216.1 (117.6)	229.6 (126.3)	188.8 (109.6)

Data reported as means (standard error of the mean, SEM) per group. ** $p<0.01$ difference Urban (Games-Howell test).
